# Relationship Between Socioeconomic Variables and Obesity in Korean Adolescents

**DOI:** 10.2188/jea.JE20100099

**Published:** 2011-07-05

**Authors:** In-Hwan Oh, Youngtae Cho, So-Youn Park, Changmo Oh, Bong-Keun Choe, Joong-Myung Choi, Tai-Young Yoon

**Affiliations:** 1 Department of Preventive Medicine, Kyung Hee University School of Medicine, Seoul, Korea; 2 School of Public Health, Seoul National University, Seoul, Korea; 3 Department of Internal Medicine, Kyung Hee University School of Medicine, Seoul, Korea

**Keywords:** socioeconomic factors, obesity, adolescent, Korea, subjective social status

## Abstract

**Background:**

Despite the importance of obesity and its association with socioeconomic status, little is known about this condition in Korean adolescents. We examined the relationship between obesity in Korean adolescents and several socioeconomic variables and compared the association of obesity with conventional and subjective indicators of socioeconomic status.

**Methods:**

The study comprised 60 643 Korean adolescents aged 12 to 18 years who participated in the 2007 Korea Youth Risk Behavior Web-Based Survey. The dependent variable, obesity, and the independent variables of parental education levels, family affluence scale, subjective family economic status, and subjective school achievement were collected by using a self-administered anonymous questionnaire. Data on behavioral and psychological characteristics were also collected and used as confounding factors. Multivariate logistic regression was conducted to identify associations between socioeconomic status and obesity.

**Results:**

In the descriptive analysis, adolescents with low parental education, low family affluence level, low subjective family economic status, and low subjective school achievement were more likely to be obese. However, after controlling for other risk factors in multivariate analysis, only the associations with subjective family economic status and subjective school achievement remained statistically significant.

**Conclusions:**

Our results provide further evidence that the prevalent pattern of obesity in Korean adolescents—i.e., the inverse relationship between obesity and socioeconomic status—is similar to that in developed countries. In addition, these findings support the hypothesis that, as compared with objective socioeconomic status, subjective social status is more closely related to obesity.

## INTRODUCTION

The prevalence of obesity in adolescents has increased worldwide.^1^^,^^2^ In the United States, the prevalence of obesity tripled during 1970–2000.^3^ Similarly, the prevalence of obesity among children and adolescents in Korea increased by 70% between 1997 and 2005.^4^ Genetics, environment, and lifestyle have all been discussed as possible causes of obesity. Socioeconomic variables are also known to increase the risk of obesity.^5^^–^^8^ However, the relationship between socioeconomic variables and obesity varies with the state of development of a particular country and the sex of the study subjects. For example, the prevalence of obesity is higher in females of high socioeconomic status (SES) in nations with a low human development index (HDI). In high-HDI nations, however, the prevalence of obesity is higher in females of low SES.^7^ Commonly, such differences are mainly found in females and are much less frequent in males. In one study of Korean adults, the odds ratio (OR) for obesity was lower in highly educated females than in others, but increased with income level in males.^8^

Although differences in health status and behavior associated with SES have been widely described in adults and children, findings in adolescents have been equivocal.^9^^,^^10^ This uncertainty may be partially due to the system used to measure SES in adolescents.^11^ Conventionally, the status of adolescents is viewed to be the same as the status of their parents. Therefore, parental occupation, education, and household income variables have been used as the SES measurements for adolescents.^9^^,^^10^ Torre et al investigated the parental education level of participants and found an association between high parental education and high extracurricular physical activity.^12^ However, there are 2 major flaws in such an approach. First, the adolescents often do not know the actual SES of their parents. Moreover, the response rate of low-SES children was lower than that of other socioeconomic groups.^13^^,^^14^ To overcome this problem, the family affluence scale (FAS) has been suggested. FAS comprises 4 questions that can be answered easily by adolescents.^10^^,^^13^^,^^15^ It can also be used to measure the level of material deprivation in adolescents.^16^ In addition, it has been pointed out that, unlike children, adolescents are in the midst of maturation. Therefore, some critics have maintained that an adolescent’s own status is more important than that of his/her parents, and, as a result, some studies have measured the subjective social status of adolescents.^17^^,^^18^ Piko et al measured student subjective social status by asking, “How would you rate your family’s socioeconomic status?”^17^ Goodman et al measured the subjective social status of adolescent within society and schools.^18^ This controversy regarding the appropriate indicator is related to the debate over the origin of health inequality. If material deprivation is important in the development of health inequality, then objective SES indicators are likely to be related to health outcomes. However, if psychosocial stress is important in the development of health inequality, subjective social status indicators will be more closely related to health outcomes.^19^

While the relationship between adolescent obesity and SES has been investigated in many studies conducted in other regions,^3^^,^^6^^,^^20^^,^^21^ studies of Korean adolescents have studied unrepresentative samples of adolescents and included only conventional socioeconomic variables in the analysis. Thus, the findings have been controversial.^22^^,^^23^ For example, a study conducted in 6 schools found that low parental education was a significant risk factor for obesity.^22^ On the other hand, a study that used household health insurance premiums as a measure of SES reported that high SES was a risk factor for obesity.^23^

We analyzed a nationally representative sample dataset to assess the extent to which socioeconomic variables (FAS, conventional and subjective measure of SES) were associated with obesity in Korean adolescents.

## METHODS

We used data from the 2007 Korea Youth Risk Behavior Web-based Survey (KYRBWS), which was administered by the Korea Center for Disease Control and Prevention (KCDC) from September through November 2007. The 2007 KYRBWS is a self-administered anonymous online survey comprising 129 questions in 13 categories, including obesity, physical activity, and health equity.^24^ In the 2007 KYRBWS, 78 834 students from 400 middle schools and 400 high schools were randomly selected. Each student was randomly assigned a unique identification number, which they used to log into the survey webpage in the computer room of their school. Before they began the questionnaire, an item asked potential respondents to electronically indicate whether they agreed to participate or not. Those who declined to participate did not proceed further. A total of 74 698 students agreed to participate (response rate: 94.8%). The study data are publically available from KCDC and do not have personal or school identifiers.^24^^–^^26^ Accordingly, 73 836 students aged 12 to 18 years were extracted for this study. Students who did not mark their body weight or height (1177 adolescents) and 7630 students who did not answer questions regarding their parental educational level were excluded. In addition, 4386 students who were underweight according to Korean growth standards were also excluded.^4^^,^^24^ Ultimately, data from 60 643 adolescent students (31 632 males and 29 011 females) were analyzed in this study.

Paternal and maternal education level, FAS, subjective family economic status, and subjective school achievement were used as socioeconomic variables. Parental education level has been widely used as a conventional measure of SES.^10^^,^^12^^,^^17^^,^^20^^,^^28^ In the present study, FAS was used as a more accurate proxy for parental SES and to assess material deprivation of the respondents. Subjective family economic status and subjective school achievement were used as indicators of adolescent subjective social status.^17^^,^^18^^,^^20^^,^^21^ In particular, subjective family economic status was used to measure the perceived SES of the family in society, and subjective school achievement was used to measure the personal social position in school.^6^^,^^17^^,^^18^^,^^20^^,^^21^

The FAS consists of 4 questions: Does your family own a car? No (0), Yes (1), Yes, 2 or more (2); Do you have your own bedroom? No (0), Yes (1); During the past year, how many times did you travel on holiday with your family? None (0), Once (1), Twice or more (2); and How many computers does your family have? None (0), one (1), 2 or more (2). Based on the sum of the scores for these items, students were classified into a low (0–3), middle (4–5), or high group (6–7).^10^^,^^15^^,^^24^ Subjective family economic status was evaluated by the question, “What do you perceive the economic status of your family to be?” and subjective school achievement was evaluated by the question, “What do you believe your school achievement to be?” The responses to these questions were evaluated by applying 5 grades, from low to high.^20^^,^^24^

BMI was evaluated based on self-reported body weight and height. Students were classified as obese if they were at or above the 95th percentile of sex-specific BMI for their age in the 2007 Korea National Growth Chart or had a BMI of 25 or higher.^4^^,^^24^^,^^27^

To adjust for the effects of other obesity risk factors, behavioral and psychological factors were considered.^29^^–^^33^ In the model, moderate physical activity (MPA), vigorous physical activity (VPA), sedentariness, body weight control, inadequate body weight control, smoking habit, and habitual consumption of breakfast, soft drinks, milk, fast food, and fruit were evaluated as behavior factors.^29^^–^^31^ Perceived stress and depressed mood were assessed as psychological factors.^32^^,^^33^ Participants were categorized into 2 groups, mainly on the basis of KYRBWS guidelines.^24^ For example, students who had not smoked during the past month and who did not make an effort to control their body weight during the past year were placed in the nonsmoking group and non-weight control group, respectively. Subjects who participated in 30 or more minutes of MPA for 5 or more days per week were placed into the MPA group, while those reporting 20 or more minutes of VPA 3 or more days per week were placed into the VPA group. The sedentary group included those who spent their leisure time sitting 3 or more hours a day on weekdays during the previous week. Participants who consumed fruit or soft drinks once or more a day during the previous week were included in the consumption group. Subjects who ate breakfast 3 or more times during the previous week were included in the breakfast consumption group. The fast-food consumption group included those who ate fast foods such as pizza, hamburgers, and fried chicken once or more in the previous week. A participant who drank milk 2 times per day during the previous week was placed into the high consumption group. Adolescents who fasted, vomited after meals, went on single-food diets, or took drugs for weight loss without a prescription were included in the inadequate body weight control group.

The adolescents who felt severe stress, as determined by their answer to the question, “How much stress do you usually feel?”, were placed in the perceived stress group. Subjects who responded affirmatively to the question, “Did you feel unhappiness and desperation that disturbed your normal life for at least two weeks during the past year?”, were included in the depressed mood group.

Rates of obesity were calculated with respect to the socioeconomic variables, and multivariate logistic regression analyses were conducted to evaluate school grade level, behavioral, and psychological factors in addition to parental education levels (model 1). In model 2, FAS replaced parental education levels. In model 3, parental education levels were replaced by subjective social status, and model 4 included all socioeconomic variables. Because some studies observed sex differences in obesity, and given that the inverse association between SES and obesity is predominantly found in females,^6^^–^^8^^,^^22^ the analyses were stratified by sex. Statistical analysis was performed using SAS version 9.1 (SAS Institute, Inc. Cary, NC, USA). To reflect the characteristics of the stratified cluster sampling design of KYRBWS, the ‘proc surveyfreq’ and ‘proc surveylogistic’ procedures were used. The level of statistical significance was set at 0.05.

## RESULTS

Table 1 shows the prevalence of obesity by grade level and SES characteristics. The overall rate of obesity was 9.3% (data not shown), and the rate of obesity among males (12.8%) was higher than that among females (5.5%). The rate increased with school grade from 7.4% to 17.6% in males and from 5.8% to 12.9% in females. In both sexes, obesity rates were higher among adolescents with low SES (as determined by parental education level), low FAS, and low subjective social status.

**Table 1.** Prevalence of obesity according to selected socioeconomic and demographic characteristics, by sex

	Male			Female		
		
	No. of Subjects	Obesity (%)	SE	No. of Subjects	Obesity (%)	SE
Total	31 632	12.8	0.2	29 011	5.5	0.2
Grade level						
7	5835	7.4	0.4	4989	5.8	0.3
8	5485	10.7	0.4	5034	7.5	0.3
9	5334	12.7	0.6	5175	9.2	0.4
10	5318	13.8	0.6	5133	9.6	0.4
11	4933	16.2	0.6	4440	12.2	0.5
12	4727	17.6	0.6	4240	12.9	0.5
Paternal education						
Middle school ​ or less	2476	15.5	0.9	2283	8.4	0.7
High school	12 667	13.8	0.4	12 568	6.1	0.3
College or higher	12 011	11.8	0.4	11 287	4.5	0.2
Unknown	4478	11.9	0.5	2873	5.7	0.6
Maternal education						
Middle school ​ or less	2702	16.0	0.8	2705	9.1	0.7
High school	15 847	13.4	0.3	16 351	5.4	0.2
College or higher	8071	11.6	0.5	7145	4.8	0.3
Unknown	5012	11.5	0.6	2810	5.5	0.6
Family affluence scale						
Low	9727	10.1	0.3	9101	6.5	0.3
Middle	14 731	9.4	0.3	13 693	5.4	0.2
High	7174	8.4	0.3	6217	4.5	0.2
Subjective family economic status						
Low	1410	16.6	1.1	1236	11.0	1.1
Low-middle	5158	14.2	0.5	5444	7.1	0.5
Middle	15 105	12.4	0.3	14 764	5.2	0.2
High-middle	7857	12.8	0.5	6255	4.4	0.3
High	2102	10.4	0.8	1312	4.7	0.6
Subjective school achievement						
Low	3424	15.3	0.7	2832	8.2	0.7
Low-middle	7448	13.9	0.5	7224	6.7	0.4
Middle	8632	12.3	0.5	7904	4.9	0.3
High-middle	8106	12.5	0.5	7627	5.2	0.3
High	4022	10.3	0.5	3424	3.2	0.3

SE: standard error. To reflect the stratified cluster sampling design of the 2007 Korea Youth Risk Behavior Web-based Survey, obesity prevalence was calculated by using the “survey frequency procedure” of SAS version 9.1.

The adjusted relationship between socioeconomic variables and obesity in males is shown in Table 2. In models 1 and 2, when school grade level, behavioral, and psychological factors were adjusted for, neither parental education level nor FAS significantly changed the probability of obesity. In model 3, there was an inverse relationship between obesity and subjective school achievement (middle, OR: 0.79, 95% CI: 0.68–0.92; high-middle, 0.81, 0.70–0.95; high, 0.69, 0.59–0.82). In the full model (model 4), only the OR for subjective school achievement remained significant, and an inverse relationship was observed (middle: 0.79, 0.68–0.92; high-middle: 0.82, 0.71–0.95; high: 0.71, 0.60–0.83).

**Table 2.** Adjusted and weighted ORs of obesity among Korean male adolescents (*n* = 31 632)

	Model 1^a^		Model 2^b^		Model 3^c^		Model 4^d^	
				
	OR	95% CI	OR	95% CI	OR	95% CI	OR	95% CI
Paternal education (Middle school or less)								
High school	1.03	0.88–1.21					1.03	0.88–1.21
College or higher	0.94	0.79–1.11					0.94	0.79–1.11
Unknown	1.13	0.91–1.40					1.12	0.90–1.38
Maternal education (Middle school or less)								
High school	0.93	0.80–1.09					0.93	0.80–1.09
College or higher	0.91	0.76–1.08					0.90	0.76–1.08
Unknown	0.86	0.69–1.06					0.84	0.68–1.04
Family affluence scale (Low)								
Middle			0.92	0.79–1.06			0.94	0.81–1.09
High			0.95	0.81–1.11			1.00	0.85–1.17
Subjective family economic status (Low)								
Low-middle					0.88	0.73–1.06	0.88	0.73–1.06
Middle					0.85	0.71–1.01	0.86	0.72–1.02
High-middle					0.96	0.79–1.16	0.98	0.81–1.19
High					0.82	0.64–1.05	0.84	0.65–1.08
Subjective school achievement (Low)								
Low-middle					0.89	0.78–1.02	0.89	0.78–1.02
Middle					0.79	0.68–0.92	0.79	0.68–0.92
High-middle					0.81	0.70–0.95	0.82	0.71–0.95
High					0.69	0.59–0.82	0.71	0.60–0.83

Δ −2 Log L	−64 286.50		−63 671.20		−63 816.10		−66 586.80	

OR: odds ratio, CI: confidence interval. To reflect the stratified cluster sampling design of the 2007 Korea Youth Risk Behavior Web-based Survey, ORs and 95% CIs were constructed using the “survey logistic procedure” of SAS version 9.1.

^a^Including the following variables: grade level of student, vigorous physical activity, moderate physical activity, sedentariness, fruit consumption, milk consumption, fast-food consumption, soft drink consumption, routine consumption of breakfast, weight control behavior, inadequate weight control behavior, smoking, perceived stress and depressed mood, paternal and maternal education.

^b^Including the variables in model 1 plus family affluence scale instead of parental education.

^c^Including the variables in model 1 plus subjective family economic status and subjective school achievement instead of parental education.

^d^Including the variables in model 1 plus family affluence scale, subjective family economic status, and subjective school achievement.

Table 3 shows the adjusted relationships between the socioeconomic variables and obesity in females with respect to school grade level and physical, dietary, and psychological factors. In model 1, paternal education of college or higher (OR 0.73, 95% CI: 0.55–0.96) and a maternal education level of high school decreased the probability of obesity (0.72, 0.58–0.89). In addition, in model 2, obesity in females was associated with low FAS (middle: 0.76, 0.60–0.95; high: 0.78, 0.60–0.997). In model 3, subjective social status was inversely associated with obesity. In the full model (model 4), high subjective family economic status and high subjective school achievement decreased the odds of obesity. In contrast, FAS and parental education level (except a maternal education level of high school; OR 0.75, 0.60–0.93) did not affect ORs for obesity in female adolescents.

**Table 3.** Adjusted and weighted ORs of obesity among Korean female adolescents (*n* = 29 011)

	Model 1^a^		Model 2^b^		Model 3^c^		Model 4^d^	
				
	OR	95% CI	OR	95% CI	OR	95% CI	OR	95% CI
Paternal education (Middle school or less)								
High school	0.92	0.74–1.16					0.96	0.76–1.20
College or higher	0.73	0.55–0.96					0.78	0.59–1.03
Unknown	0.99	0.69–1.41					0.96	0.68–1.36
Maternal education (Middle school or less)								
High school	0.72	0.58–0.89					0.75	0.60–0.93
College or higher	0.81	0.62–1.06					0.86	0.65–1.13
Unknown	0.77	0.56–1.07					0.78	0.56–1.08
Family affluence scale (Low)								
Middle			0.76	0.60–0.95			0.85	0.67–1.08
High			0.78	0.60–0.997			0.97	0.74–1.26
Subjective family economic status (Low)								
Low-middle					0.68	0.52–0.89	0.70	0.54–0.91
Middle					0.56	0.44–0.70	0.60	0.48–0.75
High-middle					0.54	0.42–0.69	0.59	0.46–0.75
High					0.65	0.44–0.94	0.69	0.47–1.01
Subjective school achievement (Low)								
Low-middle					0.84	0.68–1.04	0.84	0.68–1.04
Middle					0.64	0.51–0.81	0.64	0.51–0.81
High-middle					0.70	0.56–0.87	0.70	0.57–0.87
High					0.46	0.34–0.61	0.46	0.35–0.62

Δ −2 Log L	−26 274.53		−24 547.96		−29 393.81		−31 110.01	

OR: odds ratio, CI: confidence interval. To reflect the stratified cluster sampling design of the 2007 Korea Youth Risk Behavior Web-based Survey, ORs and 95% CIs were constructed using the “survey logistic procedure” of SAS version 9.1.

^a^Including the following variables: grade level of student, vigorous physical activity, moderate physical activity, sedentariness, fruit consumption, milk consumption, fast-food consumption, soft drink consumption, routine consumption of breakfast, weight control behavior, inadequate weight control behavior, smoking, perceived stress and depressed mood, paternal and maternal education.

^b^Including the variables in model 1 plus family affluence scale instead of parental education.

^c^Including the variables in model 1 plus subjective family economic status and subjective school achievement instead of parental education.

^d^Including the variables in model 1 plus family affluence scale, subjective family economic status, and subjective school achievement.

## DISCUSSION

This study used data from the 2007 KYRBWS to investigate the relationships between socioeconomic variables and obesity in Korean adolescents. The prevalence of obesity rose with increasing school grade level and was higher in male adolescents. These results are similar to those of the 2005–2006 HBSC study, despite differences in the definition of obesity.^34^^,^^35^

In this nationally representative sample of Korean adolescents, the prevalence of obesity was higher in adolescents with low SES, as defined by parental education levels, FAS, subjective economic status of the family, and subjective school achievement. Moreover, as SES increased, the prevalence of obesity decreased in both sexes. In multivariate logistic regression adjusted for behavioral and psychological factors, high subjective social status continued to be associated with a lower OR for obesity in both sexes. Also, because SES might influence these behavioral and psychological factors, there is the potential of over-controlling in the multivariate logistic regression model. Therefore, the association between SES and obesity may be underestimated.^28^^,^^36^ These findings support the hypothesis that the pattern of obesity prevalence among Korean adolescents mirrors that in developed countries, in which obesity prevalence is inversely related to SES.^22^ In the HBSC study, which included 41 more-developed countries, 18 countries showed a negative relationship between SES and obesity, while 4 (Greenland, Latvia, Turkey, and Russia) showed a positive relationship between SES and obesity.^15^ As the per capita gross domestic product (GDP) in Korea was 21 653 dollars in 2007 and the per capita GDPs of the aforementioned 4 countries were lower than that of Korea,^37^^,^^38^ it was expected that the distribution of obesity prevalence in Korea would show the pattern found among other developed countries. A national-scale investigation of a representative group of Korean women reported a similar decrease in the prevalence of obesity with rising education level.^8^

In the study of health equity in adolescents, measurement methods have been debated.^9^^,^^10^^,^^13^ In this study, we used FAS and subjective social status indicators as well as parental education level, which is a conventional measure of SES. In the logistic regression model, parental education level and FAS were not associated with obesity in males. In females, FAS was related to obesity (in model 2). However, when subjective social status indicators were included (model 4), the effect of FAS disappeared. Instead, subjective social status indicators, particularly subjective school achievement, had an effect on the OR for obesity when subjective social status indicators were considered (model 3). This was also true for the full model (model 4). Therefore, subjective social status indicators may be more appropriate for measuring adolescent SES, at least in the case of obesity. These findings support the observation that school achievement is associated with obesity^39^^,^^40^ and the hypothesis that self-recognized or acquired social status is more important in adolescents, as they are in the process of changing from children to adults.^17^^,^^20^ The results also lend support to the hypothesis that the prevalence of obesity is more closely associated with subjective social status indicators than with objective SES factors.^28^ Goodman et al measured objective SES (household income and parental education level) and subjective social status. They also measured subjective social status using 2 scales: family social status in American society and social status of the subjects at school. They found that social status at school and objective SES were significantly related to obesity in separate models; however, when they considered subjective and objective SES variables together, only subjective social status at school remained significantly associated with obesity. Decreasing subjective social status at school was associated with 16% of the increase in the OR for obesity. They proposed that while objective SES affects obesity directly, objective SES affects obesity via subjective social status. Moreover, possible mediators of subjective social status include the limbic system, depression, and social isolation.^28^

Inaccurate measurement of parental SES has been pointed out.^9^^,^^10^^,^^13^^,^^14^ When adolescents were asked about their parents’ occupation or education, the rate of nonresponse/inaccurate response ranged from 10% to 40%, depending on the study.^14^^,^^41^^,^^42^ Korean adolescents were not an exception. In this study, 12.1% and 12.9% of adolescents did not know their paternal and maternal education level, respectively. Thus, parental education may not be an accurate or reliable measure of SES in Korea when assessed by adolescent subjects.

This study also found that the relationship between SES and obesity was more significant in female adolescents than in males. These results are analogous to those reported in studies of adolescents in the United States and Finland, in which the socioeconomic disparity of female obesity was greater than that of males. This was also mirrored in findings among Korean adults.^6^^,^^8^^,^^43^ Although various causes have been suggested for such sex differences, sex-specific attitudes concerning body weight and different methods of controlling body weight have been suggested. Of particular importance is the fact that, as society develops, negative attitudes about obesity tend to be stronger in females than in males.^8^ The social perspective toward obesity is also stricter in females than in males. As a result, females quickly adjust their lifestyle to maintain a slimmer body shape. However, changes in lifestyle, eg, maintaining a healthy diet and appropriate exercise, can increase expense; therefore, the prevalence of obesity is higher in females with lower SES.^7^^,^^8^^,^^43^^,^^44^

Regarding the limits of this study, several points warrant discussion. First, because this was a cross-sectional study, causality cannot be inferred. For example, in female adolescents who participated in VPA, the OR of obesity increased unexpectedly (data not shown). This suggests that adolescents start to undertake body weight control efforts, or to perform VPA, in response to obesity. Nonetheless, an association between subjective social status and obesity was observed in a cohort study. In that study of female adolescents, low subjective social status was related to a high increase in BMI 2 years later.^21^ However, longitudinal follow-up studies must be performed to investigate whether such a relationship exists in Korean adolescents. Another limitation is that this study excluded underweight adolescents, as well as those who did not report their BMI information. These 2 groups probably have their own distinctive features, but both were excluded from the analysis. These types of shortcomings may have occurred with the KYRBWS itself. According to the 2007 Korean society index, middle school and high school enrollment rates were 96.0% and 91.3%, respectively.^45^ Therefore, the relationship between obesity and SES in 4% to 9% of adolescents could not be determined, which hinders our overall understanding of the obesity characteristics of Korean adolescents. The inaccuracy of the self-administered questionnaire may also be seen as a limitation. Although, in a previous study, self-reported BMI values were relatively highly correlated with actual BMI (*r* = 0.92),^21^ adolescents might overestimate their height and underestimate their body weight. Another issue is parental BMI. Although parental obesity could have an effect on obesity in adolescents, due to genetics or lifestyle,^42^ this study did not consider those factors. Similarly, although we included possible confounding factors, the questionnaire did not measure all confounding factors, such as fat consumption and calorie intake. In addition, depressed mood and stress perception were measured by only 1 question and were not standardized or validated. As a result, there are concerns as to whether the confounding factors were adequately and correctly considered.

In spite of these limitations, this is the first study to analyze the association between SES and obesity among Korean adolescents using a nationally representative sample. The prevalence of obesity was high in males and in students in higher grade levels. The OR for obesity also showed an inverse relationship with SES, similar to the pattern in other developed countries. Among socioeconomic variables, subjective social status variables were significant, which supports the hypothesis that subjective social status is more strongly associated with obesity. However, because this was a cross-sectional study, we are unable to identify the mechanism that underlies development of obesity. Therefore, additional studies are needed to investigate the socioeconomic factors and pathways that are related to the development of obesity.
